# Gibberellin promotes theanine synthesis by relieving the inhibition of CsWRKY71 on *CsTSI* in tea plant (*Camellia sinensis*)

**DOI:** 10.1093/hr/uhae317

**Published:** 2024-11-18

**Authors:** Fen Xiang, Yi Su, Lingyun Zhou, Cuiting Dai, Xuan Jin, Hongyan Liu, Weigui Luo, Wenbo Yang, Wei Li

**Affiliations:** Tea Research Institute, Hunan Academy of Agricultural Science, Changsha 410125, China; Hunan Tea Plant and Tea Processing Scientific Observation Experimental Station of the Ministry of Agriculture, Changsha 410125, China; Hunan Provincial Key Laboratory of Phytohormones and Growth Development, Hunan Agricultural University, Changsha 410128, China; Tea Research Institute, Hunan Academy of Agricultural Science, Changsha 410125, China; Hunan Tea Plant and Tea Processing Scientific Observation Experimental Station of the Ministry of Agriculture, Changsha 410125, China; Tea Research Institute, Hunan Academy of Agricultural Science, Changsha 410125, China; Hunan Tea Plant and Tea Processing Scientific Observation Experimental Station of the Ministry of Agriculture, Changsha 410125, China; Hunan Provincial Key Laboratory of Phytohormones and Growth Development, Hunan Agricultural University, Changsha 410128, China; Tea Research Institute, Hunan Academy of Agricultural Science, Changsha 410125, China; Hunan Tea Plant and Tea Processing Scientific Observation Experimental Station of the Ministry of Agriculture, Changsha 410125, China; Lushan Botanical Garden, Chinese Academy of Science, Jiu Jiang 332900, China; Tea Research Institute, Hunan Academy of Agricultural Science, Changsha 410125, China; Hunan Tea Plant and Tea Processing Scientific Observation Experimental Station of the Ministry of Agriculture, Changsha 410125, China; Tea Research Institute, Hunan Academy of Agricultural Science, Changsha 410125, China; Hunan Tea Plant and Tea Processing Scientific Observation Experimental Station of the Ministry of Agriculture, Changsha 410125, China; Hunan Provincial Key Laboratory of Phytohormones and Growth Development, Hunan Agricultural University, Changsha 410128, China

## Abstract

Theanine is a crucial indicator of tea quality, and its significance is closely tied to the economic value of tea. There have been many reports on the regulation mechanism of theanine synthesis and accumulation, but the mechanism by which gibberellin regulates theanine synthesis in tea plants is poorly understood. Previous studies have shown that the content of theanine experiences significant changes in the growth stages of tea shoots, displaying a strong correlation with gibberellin. This study confirmed that gibberellin significantly promoted the expression of the major gene of theanine synthesis, known as *CsTSI*. Additionally, the study identified CsWRKY71 as a transcription factor that mediated the regulation by gibberellin of theanine synthesis in tea plants. CsWRKY71 was localized in the nucleus and had a typical WRKY domain. It was a member of subclass IIC and its expression was significantly suppressed following exogenous GA_3_ treatment. Further assays, such as the electrophoretic mobility shift assay, dual luciferase and asODN (antisense oligodeoxynucleotide) interference, demonstrated that CsWRKY71 significantly interacted with the promoter of *CsTSI*, which inhibited theanine synthesis by binding to the *cis*-acting element (C/T)TGAC(T/C) of the *CsTSI* promoter. Overall, the addition of exogenous gibberellin alleviated the inhibition of *CsTSI* by down-regulating the expression of *CsWRKY71*, ultimately facilitating the rapid biosynthesis of theanine. This study elucidated the molecular mechanism of CsWRKY71-mediated gibberellin regulation of theanine synthesis in tea plant. The findings not only enhance our understanding of the regulatory processes involved in theanine synthesis in tea plants, but also provide important references for maintaining the characteristics of high theanine in the tea plant.

## Introduction

Theanine is a unique non-protein amino acid found in the tea plant (*Camellia sinensis*). It constitutes almost 50% of the total free amino acids in tea leaves (dry weight), which significantly impacts the economic value of tea [1]. The functions of theanine related to food science, nutritional value, and health attributes have been extensively reported ever since its discovery in tea leaves. As a special free amino acid, theanine is easily absorbed and transported in the human body [2–4]. It can cross the blood–brain barrier and possesses a variety health-promoting effects, including anxiolytic, antitumor, neuron protection, and potential therapeutic applications in Parkinson’s disease.

Theanine is synthesized from glutamate and ethylamine by theanine synthetase mainly in tea roots [5, 6]. During the germination and rapid growth of tea shoots, nitrogen is transported and stored in the form of theanine and glutamine in the apical new shoot [7–9]. It not only serves as the main quality component in tea, but also serves as an energy source and biosynthetic precursor of variable bioactive molecules [10, 11]. Moreover, under the catalysis of other enzymes, such as glutamine synthetase, theanine can also be synthesized in tea leaves with ethylamine and glutamate as substrates [12].

The process of absorbing exogenous nitrogen and converting it into theanine in the tea plant is closely linked to the release of dormancy, germination, and growth of overwintering buds. This process is affected by external factors such as temperature, light radiation intensity, and soil nutrients, which in turn acting on the expression of related genes involved in theanine biosynthesis [12–15]. Plant transcription factors (TFs), including MYBs and WRKYs, have recently been identified as key genes in *C. sinensis* that can influence the metabolic pathways related to theanine synthesis, transport, and hydrolysis [16–19].

The WRKY family is one of the largest TF families in plants [20]. They have the ability to specifically identify and bind to *cis*-acting elements ((C/T)TGAC(T/C)) in gene promoters [21, 22]. WRKY TFs play a crucial role in regulating various bio-processes, such as plant growth, disease resistance, and secondary metabolism, through their involvement in the phytohormone signal transduction pathway [23, 24]. For example, WRKYs like WRKY71, WRKY51, and WRKY38 are key TFs that mediate gibberellin regulation of the barley *Amy32b* α-amylase gene’s expression [21, 25, 26]. WRKYs also participate in plant secondary metabolism regulation, including nitrogen uptake and conversion in many plant species [20]. For instance, up-regulation of WRKY33 causes the high expression of several genes related to nitrogen use efficiency in *Solanum melongena* [27], and WRKY40 binds to the *CsPDX2.1* promoter to regulate the hydrolysis of theanine during tea leaf withering in *C. sinensis* [18, 27]. Theanine, being an important nitrogen storage substance in tea plant nitrogen metabolism, is probably regulated by WRKYs.

In a previous study, we found that the content of gibberellin A3 (GA_3_) in tea plant was significantly correlated with the theanine content during the germination stages of tea shoots [28]. Exogenous GA_3_ resulted in the up-regulation of genes participating in theanine biosynthesis, and led to a 27% increase in theanine content in tea leaves [28]. These findings indicated that WRKYs related to the gibberellin signaling pathway might play a crucial role in the process of theanine synthesis. In this study, a transcription factor, CsWRKY71, was cloned and characterized, which was involved in the gibberellin signaling pathway in tea plant. This study not only elucidated the molecular mechanism of CsWRKY71-mediated gibberellin regulation of theanine synthesis, but also provided important references for maintaining the characteristics of high theanine in tea plant.

## Results

### Exogenous GA_3_ promotes the expression of *CsTSI* in tea roots

The main synthesis site of theanine is tender roots of tea plant, and *CsTSI* is the critical gene in the theanine synthesis pathway. The *CsTSI* from ‘Baojing Huangjincha 1’ (HJ1, a variety with high theanine content) was cloned and transferred into *Arabidopsis thaliana* for the identification of its biological function (Supplementary Data Fig. S1). To investigate the response of *CsTSI* and theanine content to gibberellin signals, tea plants were treated with a final concentration of 1 μmol L^−1^ GA_3_ to analyze the variation of *CsTSI* relative expression in tea plant. Primers are described in Supplementary Data Table S1.

**Figure 1:**
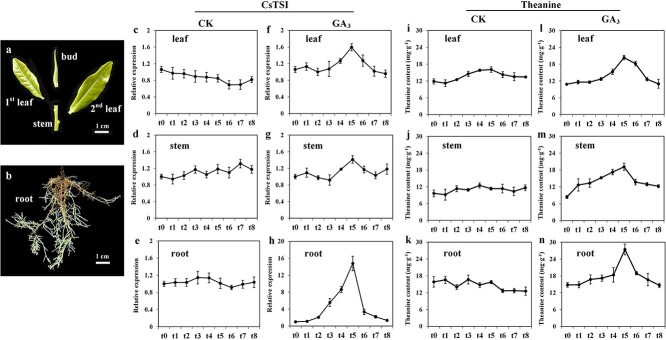
Variation of theanine content and relative expression of *CsTSI* under GA_3_ treatment in tea plants. **a**, **b** Schematic diagram of tea leaf (buds, first, and second leaves were collected as leaf samples), stem and root samples in tea seedlings after 60 days of hydroponic cultivation. **c**–**h** Relative expression of *CsTSI* in hydroponic tea seedlings treated with gibberellin at 0 μmoL L^−1^ (control, CK) and 1 μmol L^−1^. **i**–**n** Theanine content in tea seedlings treated with gibberellin at 0 μmol L^−1^ (CK) and 1 μmol L^−1^. t0 represents samples collected before GA_3_ treatment. t1–t8 represent samples collected 0.5, 1, 2, 4, 8, 16, 32, and 48 h after GA_3_ treatment. Data in **c**–**n** are presented as the mean ± standard deviation of three biological replicates.

Under the control condition, the relative expression of *CsTSI* in the tender roots, stems and leaves fluctuated slightly within 48 h, and remained stable overall (Fig. 1c–e). Under GA_3_ (1 μmol L^−1^) treatment, the expression level of *CsTSI* in roots, stems and leaves increased gradually from t0 to t5 (t0 represents samples collected before GA_3_ treatment and t1–t8 represent samples collected at 0.5, 1, 2, 4, 8, 16, 32, and 48 h after GA_3_ treatment), reaching its peak at t5, and then gradually decreased. After 48 hours (t8), its expression level returned to its pretreatment level (Fig. 1f–h). In addition, according to the relative expression level in tea plants, the up-regulation of *CsTSI* in roots was much higher than that in stems and leaves.

Under the control condition, the content of theanine in the roots, stems, and leaves of tea plants fluctuated slightly within 48 h, and remained stable overall (Fig. 1i–k). After GA_3_ treatment, the theanine content in the roots of tea plants increased gradually from t0 to t5 and reached its peak at t5 (Fig. 1l–n), then gradually decreased and returned to the level before treatment at t8. The content of theanine in the stems and leaves of tea plants was basically the same as that in roots; its content began to rise from t3 and reached the peak at t5, then gradually decreased to the level before treatment at t8. According to the range of change in theanine content, it increased the most in tea roots, and its peak value was the highest, which was consistent with the change trend of relative expression of *CsTSI*.

### Response of the promoter of theanine synthetase gene to GA

**Figure 2:**
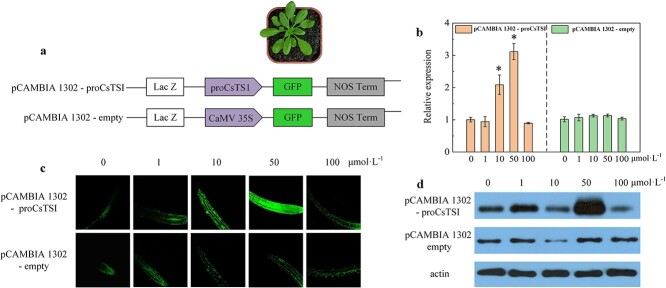
Gibberellin up-regulated the expression of downstream genes of *proCsTSI*. **a** Diagram showing replacement of CaMV35S in pCAMBIA 1302 with *proCsTSI*. **b**  *GFP* relative expression in *A. thaliana* of transgenic *proCsTSI* line and pCAMBIA 1302-empty line under GA_3_ treatments. **c** Fluorescence intensity of GFP in *A. thaliana* of transgenic *proCsTSI* line and pCAMBIA 1302-empty line under GA_3_ treatments. **d** GFP in *A. thaliana* of transgenic *proCsTSI* line and pCAMBIA 1302-empty line under GA_3_ treatments. The GA_3_ treatment concentration was 0, 1, 10, 50, and 100 μmol L^−1^. Data are presented as the means ± standard deviation of three biological replicates. ^*^P < 0.05.

The *CsTSI* gene might be directly regulated by certain TFs in the GA signal pathway, or indirectly regulated by increasing the rate of its substrate synthesis. Therefore, the heterologous transformation of the promoter of *CsTSI* was employed to analyze the issue. Primers are listed in Supplementary Data Table S1. The CaMV35S of *GFP* in pCAMBIA 1302 was replaced with the *CsTSI* promoter (*proCsTSI*, Fig. 2a). The *proCsTSI* mutant of *A. thaliana* was treated with GA_3_. The results showed that the relative expression (Fig. 2b), the fluorescence intensity and the protein of GFP (Fig. 2c) significantly increased after treatment with GA_3_ concentrations ranging from 10 to 50 μmol L^−1^. However, as the concentration of GA_3_ increased to 100 μmol L^−1^ the expression of GFP rapidly decreased (Fig. 2b–d), indicating that there was a threshold concentration for enhancing the expression of GFP, and high concentrations of GA_3_ inhibited the expression of GFP. The promoter of *GFP* in the mutant was replaced by *proCsTSI*, indicating that GA_3_ (10–50 μmol L^−1^) was conducive to the increase of *CsTSI* expression, which was beneficial to theanine biosynthesis.

### CsWRKY71 is classified in the IIc subgroup of the WRKY transcription factor family

**Figure 3:**
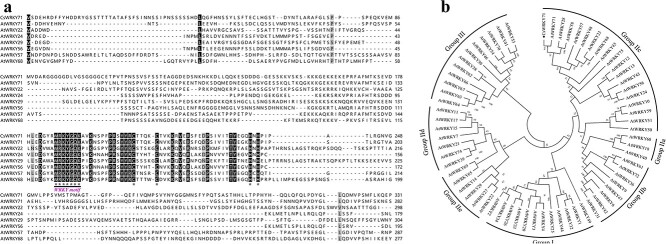
Amino acid sequence and phylogenetic analysis of CsWRKY71. **a** Amino acid sequence alignment of CsWRKY71 and AtWRKYs. Black line indicates WRKY motifs. Asterisks indicate zinc finger structures. **b** Phylogenetic analysis of CsWRKY71 and AtWRKYs*.*

Through RNA-seq analysis (NCBI under the master accession number PRJNA1138925, Fig. S2a–b), three TFs, CsWRKY71, CsERF11, and CsPBF1, related to gibberellin signal transduction were screened. Bioinformatics analysis showed that the binding sites of WRKY71 and PBF1 were found from 1350 bp upstream to the initiation codon of the *proCsTSI* (Supplementary Data Table S2). Further qPCR analysis showed that CsWRKY71 was significantly negatively correlated with the relative expression of *CsTSI* (Fig. S2c–f, Supplementary Data Table S3). Therefore, CsWRKY71 was speculated to be a WRKY family TF binding to *proCsTSI* and to participate in gibberellin signal transduction (Fig. 3a). The gene was cloned according to the Tea Plant Information Archive (TPIA). Primers are described in Supplementary Table S1. The cDNA length of *CsWRKY71* was 996 bp, encoding 332 amino acids.

According to the number of conserved WRKY domains and the type of zinc finger structure, WRKY TFs in plants are classified into three major groups (I–III, Fig. 3a). There were five subclasses (IIa–IIe) in group II. Based on the phylogenetic analysis, CsWRKY71 was a member of subclass IIc (Fig. 3b). The main function of *AtWRKY71*, a homologous gene of *CsWRKY71*, was closely related to the regulation of plant secondary metabolism, which supports the possible function of CsWRKY71, such as theanine biosynthesis, in *C. sinensis*.

**Figure 4:**
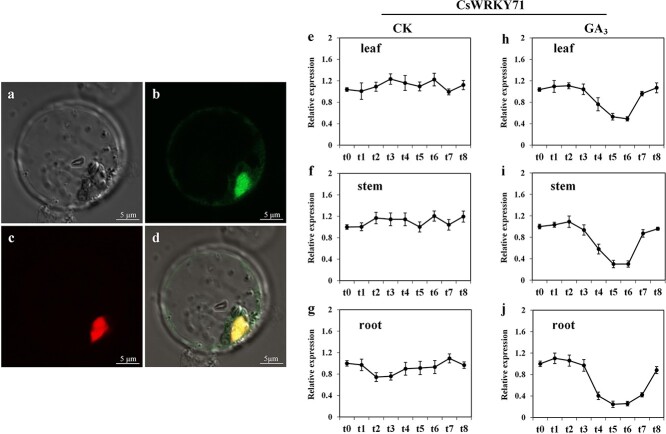
Subcellular localization of *CsWRKY71* in rice protoplast cells. **a**–**d** Protoplasts isolated from a rice marker line with OsNLS-RFP were transiently transformed with *35S::WRKY71-EGFP*. **e**–**j** Relative expression level of *CsWRKY71* response to GA_3_ at 0 μmol L^−1^ (control) and 1 μmol L^−1^ in tea leaves, stems, and roots. t0 represents samples collected before GA_3_ treatment, t1–t8 represent samples collected 0.5, 1, 2, 4, 8, 16, 32, and 48 h after GA_3_ treatment. Data in **e**–**j** are presented as the means ± standard deviation of three biological replicates.

The relative expression of *CsWRKY71* in roots, stems, and leaves after 1 μmol L^−1^ GA_3_ treatment was determined. Compared with the control, the expression of *CsWRKY71* in roots, stems, and leaves was down-regulated after GA_3_ treatment and reached the lowest level at t5 and t6, then its expression gradually returned to the same level as the control at t8 (Fig. 4e–j). Combined with the results of *CsTSI* promoter element analysis and the change trend of the relative expression of *CsTSI* under GA_3_ treatment (Fig. 1i–n), it can be further speculated that GA_3_ probably regulated the biosynthesis of theanine by depressing the *CsWRKY71* expression level. The transcriptional regulation process of WRKYs generally occurs in the nucleus. To verify the subcellular localization of CsWRKY71 expression *in vivo*, it was inserted into a pHBT-GFP plasmid and co-transfected with nuclear marker gene *NLS-RFP* into rice protoplasts. Primers are listed in Supplementary Data Table S1. As shown in Fig. 4a–d, the GFP signal fused with CsWRKY71 was mainly concentrated in the nucleus, indicating that CsWRKY71 was a typical nuclear protein with TF characteristics.

### CsWRKY71 binds directly to the *CsTSI* promoter to repress theanine biosynthesis

**Figure 5:**
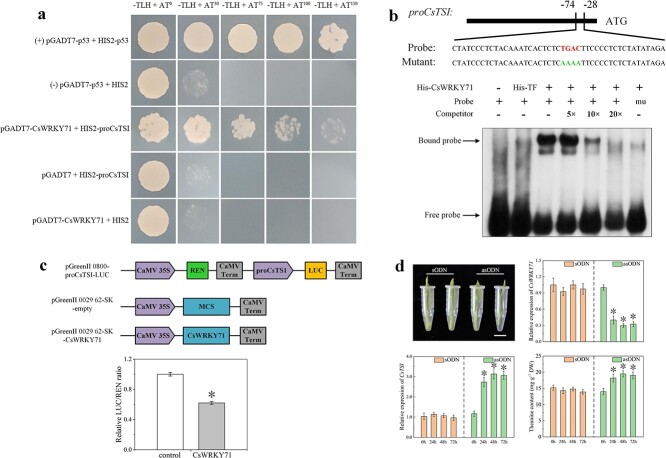
CsWRKY71 binds directly to the promoter of *CsTSI* to repress theanine biosynthesis. **a** Interaction of CsWRKY71 and the promoter of *CsTSI*, named *proCsTSI*, in yeast cells by Y1H assay. -TLH indicates Trp, His, and Leu synthetic dropout media. AT^0^–AT^150^ indicate 0–150 mmol L^−1^ 3-AT. **b** EMSA of CsWRKY71 binding to the fragment of *proCsTSI* (−74 to −28 bp). ‘Probe’ indicates DNA probes, while ‘Mutant’ and ‘mu’ indicate mutated probes. ‘+’ indicates presence and ‘-’ indicates absence. **c** Diagrams of vector construction for dual-luciferase assay and the ratio of LUC to REN activity. **d** Suppression of CsWRKY71 in tea plants by asODN assay. Tea shoots were subjected to 20 μmol L^−1^ sODN or asODN for 24–72 h; the column diagrams represent theanine content and the relative expression of *CsWRKY71* and *CsTSI*; the scale bar represents 1 cm. Data are presented as the means ± standard deviation of three biological replicates. ^*^P < 0.05.

To analyze the interaction between CsWRKY71 and *proCsTSI*, the yeast-one-hybrid (Y1H) assay was employed to verify the potential binding activity of them. Primers used in this assay are described in Supplementary Data Table S1. As shown in Fig. 5a, at a 3-AT concentration of 75 mmol L^−1^ the positive control and pGADT7-CsWRKY71 showed better colony growth, while the negative control showed sterile colony growth. These data indicated an interaction between CsWRKY71 and *proCsTSI* regulating the expression of downstream gene *CsTSI*.

Previous research showed that WRKYs directly bound to the W-box in the target gene promoter to regulate the expression of downstream genes. In this study, the electrophoretic mobility shift assay (EMSA) was applied to further verify the interaction between CsWRKY71 and *proCsTSI*. A probe (−74 to −28 bp) containing the *cis*-acting element ((C/T)TGAC(T/C)) of *proCsTSI* was cloned and His-CsWRKY71 and His-TF (trigger factor) proteins were purified from *Escherichia coli* Rosetta BL21 strain (Fig. 5b). Primers used in this assay are described in Supplementary Data Table S1. Results showed that His-CsWRKY71 was capable of recognizing and binding to the probe containing the W-box ((C/T)TGAC(T/C)) of *proCsTSI*. The binding signal was weakened with increasing concentrations of cold competitor. No band was observed when His-TF was added in place of His-CsWRKY71 (Fig. 5b). The dual-luciferase assay was further performed to analyze the sequence-specific interaction between CsWRKY71 and *proCsTSI*. The recombinant protein pGreenII 0800-*proCsTSI* (reporter gene) and the fusion protein containing *CsWRKY71* (effector gene) were transiently expressed in tobacco leaves. Primers are described in Supplementary Data Table S1. The co-transformation of them led to a significantly lower Luc/Ren ratio than control (Fig. 5c), indicating that CsWRKY71 could bind to *proCsTSI* and inhibit the expression of the reporter gene. To verify the negative role of CsWRKY71 in theanine biosynthesis, tea shoots of HJ1 were treated with CsWRKY71-specific sense oligonucleotides (sODNs) or antisense oligonucleotides (asODNs) for 72 h to transiently knock down *CsWRKY71*. Primers are listed in Supplementary Data Table S1. Results showed that expression of CsWRKY71 was significantly suppressed, while *CsTSI* relative expression and theanine content rapidly increased in tea shoots. These data verified that CsWRKY71 binds to the promoter of *CsTSI* to regulate theanine biosynthesis in tea plants.

## Discussion

Theanine, as the most abundant amino acid component in tea, is regulated by various factors. A previous study revealed a significant positive correlation between the variation of theanine and gibberellin contents during growth stages from bud germination to one bud with four leaves in ‘Baojing Huangjincha 1’ (HJ1) [28]. Subsequent research showed that after the tea plant roots were treated with exogenous GA_3_, *CsTSI* expression and theanine content were up-regulated in the roots, stems, and leaves of hydroponic tea seedlings (Fig. 1) [28]. However, the molecular mechanism of gibberellin affecting theanine synthesis remained unclear. In this study, the 35S promoter of *GFP* in pCAMBIA1302 was replaced with *proCsTSI* and transferred into *A. thaliana* (Fig. 2). The relative expression and fluorescence intensity of GFP increased significantly after GA_3_ (50 μmol L^−1^) treatment, indicating that the expression of *CsTSI* could be up-regulated by GA_3_ through its signal transduction pathway (Fig. 2).

WRKYs constitute one of the largest TF families in plants, and they often play key roles in both the repression and activation of important plant processes [21, 29]. For example, OsWRKY71 was identified as a key regulator mediating the crosstalk of gibberellin and abscisic acid in aleurone cells of rice, and specifically repressed GA-induced *Amy32b* α-amylase promoter activity [26]; CsWRKY57like was verified to activate the expression of genes related to methylated EGCG biosynthesis by binding to the *cis*-acting element (C/T)TGAC(T/C) on their promoters in tea plants [30]. Based on the results of RNA-seq (NCBI accession number: PRJNA1138925) and bioinformatic analysis of *proCsTSI*, a member of the WRKY family, named CsWRKY71, might be involved in the regulation of theanine biosynthesis by gibberellin in tea plant [31–33].

**Figure 6:**
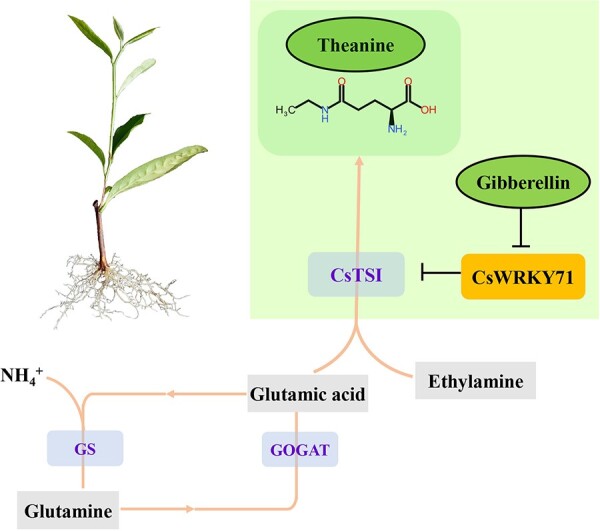
Working model of the regulatory functions of CsWRKY71 in theanine biosynthesis. CsWRKY71 binds with the promoter of *CsTSI* to inhibit the expression of downstream *CsTSI*. Gibberellin (exogenous GA_3_ concentration 1 μmol L^−1^) suppresses CsWRKY71 to relieve the inhibition of *CsTSI* and promote theanine synthesis.

In this study, CsWRKY71 was identified as a typical TF of the WRKY family, classified in the same clade with AtWRKY71 and OsWRKY71/OsWRKY51 in the group IIc subfamily (Fig. 3). It has been reported that OsWRKY71 and OsWRKY51, highly homologous to CsWRKY71, act as transcriptional repressors of GA signaling in aleurone cells to block the expression of the α-amylase gene [25]. Exogenous gibberellin decreases the steady-state mRNA level of *OsWRKY71* and destabilizes the OsWRKY71-GFP fusion protein, which has benefits for the expression of *Amy32b* in rice [25]. These findings support the notion that CsWRKY71 mediates the regulation of theanine synthesis by gibberellin. Our results demonstrated that the function of CsWRKY71 was located in the nucleus (Fig. 4), which was consistent with the localization of TFs. Both EMSA and the dual-luciferase assay showed that CsWRKY71 negatively regulated *CsTSI* by recognizing and binding to the W-box (C/T)TGAC(T/C) of *proCsTSI* (Fig. 5). Furthermore, the asODN assay was used to inhibit the expression of CsWRKY71, resulting in a significant increase in theanine content in tea shoots. Exogenous GA_3_ also inhibited the expression of CsWRKY71 (Fig. 4), and significantly increased theanine content and *CsTSI* expression in tea plants, especially in the roots (Fig. 1). These results indicated that, at a certain concentration (1 μmol L^−1^), gibberellin promoted theanine synthesis in the tea plant by relieving the inhibition of CsWRKY71 on *CsTSI* (Fig. 6).

This study provides a preliminary understanding of the mechanism through which gibberellin regulates theanine synthesis in the tea plant via its signal pathway, and offers a theoretical reference for improving tea quality and creating tea varieties with high theanine content. Additionally, the application of gibberellin holds practical significance for improving the yield of tea shoots, delaying the period of tea picking and improving the economic value of tea.

## Materials and methods

### Plant material and treatments

Cuttings from tea variety ‘Baojing Huangjincha 1’ (HJ1) were propagated for new tea plants, reaching industrial size over a period of 2 years. The contents of trace elements in nutrient solution were as follows: CuSO_4_ 5H_2_O, 0.08 g L^−1^; H_3_BO_3_, 2.5 g L^−1^; MoO_3_, 0.05 g L^−1^. During the culture stage, oxygen was supplied to the solution via a booster pump, and the nutrient solution was renewed every 7 days. The light intensity of the cultivation environment was set at 400 μmol m^−2^ s^−1^, with a light cycle of 12 h light:12 h darkness.

### Treatments

GA_3_ treatments were applied to the tea seedlings when the tender roots of the tea plant developed to 3–5 cm in length. The final concentration of GA_3_ (Sigma–Aldrich LLC, catalog # 48880) in hydroponic solution was 0 μmol L^−1^ (control) and 1 μmol L^−1^. Four repeated experiments were performed.

### Sample collection

Tender leaves (including buds, first leaves, and second leaves), stems, and roots (Fig. 1a–b) samples were collected as control samples before processing, defined as t0. Following GA_3_ treatment at 0.5 h (t1), 1 h (t2), 2 h (t3), 4 h (t4), 8 h (t5), 16 h (t6), 32 h (t7) and 48 h (t8), tender leaves, stems, and roots of tea plants were collected. The samples were frozen in liquid nitrogen after being dried with filter paper. Samples were stored at −80°C for subsequent experiments.

### Genomic DNA and total RNA extraction

The samples were ground into fine powder in liquid nitrogen. Genomic DNA and total RNA were extracted from 80–120 mg of the powder. Genomic DNA was extracted using a Plant DNA Kit (Beijing, China). Total RNA was extracted using a Plant RNA Pure Kit (Beijing, China). These kits were purchased from Tiangen company. The quality of both genomic DNA and total RNA was identified by 1% gel electrophoresis and a NanoDrop One spectrophotometer (NanoDrop, Wilmington, USA) [25].

### Quantitative PCR assay

The cDNA was diluted to 200 ng μL^−1^ for the template in the next step. The PreMix kit for quantitative PCR (qPCR) was purchased from Tiangen Biotech company (Beijing, China) and the program was run on the CFX96 real-time system (Bio-Rad, Hemel Hempstead, UK). The qPCR program was performed as follows: 5 min at 95°C, then 40 cycles of 95°C for 10 s and 62°C for 30 s [28]. β-Actin was defined as the reference for gene expression analysis. Primers in this assay are described in Supplementary Data Table S1.

### Plant transformation of *A. thaliana*, treatments and western blot

The 35S promoter of *GFP* in pCAMBIA1302 was replaced with *proCsTSI* and reconstituted into pCAMBIA1302::proCsTSI. The primer sequences are shown in Supplementary Data Table S1. pCAMBIA1302::proCsTSI was transferred into wild-type *A. thaliana* using *Agrobacterium* infection.

*Arabidopsis thaliana* plants were cultivated on MS medium to which concentrations of GA_3_ of 0, 1, 10, 50, and 100 μmol L^−1^ were added, and the GFP fluorescence intensity was observed using FV1000 laser scanning confocal microscopy (Olympus, Tokyo, Japan) after 7 days of culture.

Total protein of *A. thaliana* was collected and separated by SDS–PAGE electrophoresis. Total protein was subjected to immune reaction. After several washes, it was exposed and developed to observe the expression of the target protein in *A. thaliana*.

### Subcellular localization

To analyze the subcellular localization of *CsWRKY71*, the 35S::WRKY71-EGFP/PHBT vector was generated by transferring *CsWRKY71* into the PHBT vector, digestion sites SalI–SacII. The 35S::WRKY71-EGFP/PHBT vector was subsequently transformed into rice protoplasts. Fluorescence images were obtained using a Zeiss Axio Observer 7 (Oberkochen, Germany).

### Yeast one-hybrid assay

The Y1H assay was used to investigate the interaction between CsWRKY71 and *proCsTSI*. For prey protein construction, the *CsWRKY71* coding sequence was cloned and transformed into pGADT7 to generate the pGADT7-CsWRKY71 vector. For bait sequence construction, the *cis*-element sequence (−600 bp to start codon) of *proCsTSI* was inserted into pHIS2 to generate the pHIS2-proCsTSI vector. The linearized bait sequence pHIS2-proCsTSI was then transformed into the yeast strain Y183. The minimal inhibitory concentration of 3-AT was verified by self-activation ability testing. The prey vector pGADT7-CsWRKY71 and the bait plasmid pHIS2-proCsTSI were co-transformed into the yeast strain to detect interaction on an SD/−Leu medium supplemented with the minimal inhibitory concentration of 3-AT.

### Dual-luciferase reporter assay

To assess the interaction of CsWRKY71 with the promoter of *CsTSI*, the sequence of *proCsTSI* including the *cis*-element (from −600 bp to start codon of *CsTSI*) was cloned and inserted into the pGreen0800-LUC plasmid to provide pGreenII-proCsTSI. The coding sequence of *CsWRKY71* was inserted into the pGreenII 62-SK plasmid to construct the effector plasmid. The effector and reporter plasmids were individually transformed into *Agrobacterium* and then combined. The mixed *Agrobacterium* was then infiltrated into *Nicotiana benthamiana* leaves by injection. Empty plasmid pGreenII 62-SK was co-transformed with pGreenII-proCsTSI reporter as control [34]. Leaf samples near the injection site were collected using a 5-cm puncher 3 days after injection. The activities of firefly luciferase and *Renilla* luciferase were determined using a dual-luciferase reporter assay system (Promega, Fitchburg, USA). Primers in this assay are described in Supplementary Data Table S1.

### EMSA assay

The coding sequence of *CsWRKY71* was cloned and inserted into the pCold-TF vector (Takara, Japan) to produce His-tagged fusion proteins, and further transformed into *E. coli* Rosetta BL21 to obtain the protein His-CsWRKY71. The empty pCold-TF vector was transformed into *E. coli* strain Rosetta BL21 and was used as the negative control. Expression of His fusion proteins in *E. coli* Rosetta cells was induced and the proteins were purified according to the manufacturer’s instructions (Invitrogen, USA).

A probe containing the *cis*-acting element (C/T)TGAC(T/C) from *proCsTSI* was labeled with 3′-biotin. The same unlabeled sequences were used as competitors. The EMSA experiment was conducted following the methods described by Ma *et al*. [35]. Primers are described in Supplementary Data Table S1.

### Statistical analysis

All the experiments were performed with at least three biological replicates. The significance of differences was analyzed using Student’s *t*-test or Tukey’s multiple range test with SPSS (version 21.0; IBM Corp., Armonk, NY, USA).

## Supplementary Material

Web_Material_uhae317

## Data Availability

All relevant data in this study are provided in the article and its supplementary files.

## References

[1] Chen T, Ma J, Li H. et al. *CsGDH2.1* negatively regulates theanine accumulation in the late-spring tea plants (*Camellia sinensis* var. *sinensis*). *Hortic Res*. 2022;10:uhac245 36643747 10.1093/hr/uhac245 PMC9832843

[2] Schallier A, Vermoesen K, Loyens E. et al. L-Theanine intake increases threshold for limbic seizures but decreases threshold for generalized seizures. *Nutr Neurosci*. 2013;16:78–82 23324588 10.1179/1476830512Y.0000000033

[3] Sharma E, Joshi R, Gulati A. L-Theanine: an astounding sui generis integrant in tea. *Food Chem*. 2018;242:601–10 29037735 10.1016/j.foodchem.2017.09.046

[4] Zhu B, Qiao S, Li M. et al. Strong biosynthesis and weak catabolism of theanine in new shoots contribute to the high theanine accumulation in albino/etiolated tea plant (*Camellia sinensis*). *Beverage Plant Res*. 2023;3:1–9

[5] Liu S, Li J, Huang J. et al. New advances in genetic engineering for L-theanine biosynthesis. *Trends Food Sci Technol*. 2021;114:540–51

[6] Wei C, Yang H, Wang S. et al. Draft genome sequence of *Camellia sinensis* var. *sinensis* provides insights into the evolution of the tea genome and tea quality. *Proc Natl Acad Sci USA*. 2018;115:4151–8 10.1073/pnas.1719622115 PMC5939082 29678829

[7] Xie N, Huang X, Zhou J. et al. The R2R3-MYB transcription factor CsMYB42 regulates theanine biosynthesis in albino tea leaves. *Plant Sci*. 2023;336:111850 37648117 10.1016/j.plantsci.2023.111850

[8] Li F, Dong C, Yang T. et al. Seasonal theanine accumulation and related gene expression in the roots and leaf buds of tea plants (*Camellia sinensis* L.). *Front Plant Sci*. 2019;10:1397 31749819 10.3389/fpls.2019.01397 PMC6842895

[9] Liu Z, Wu Z, Li H. et al. L-Theanine content and related gene expression: novel insights into theanine biosynthesis and hydrolysis among different tea plant (*Camellia sinensis* L.) tissues and cultivars. *Front Plant Sci*. 2017;8:1–11 28439281 10.3389/fpls.2017.00498 PMC5383724

[10] Ku K, Choi J, Kim J. et al. Metabolomics analysis reveals the compositional differences of shade grown tea (*Camellia sinensis* L.). *J Agric Food Chem*. 2010;58:418–26 19994861 10.1021/jf902929h

[11] Liu J, Zhang Q, Liu M. et al. Metabolomic analyses reveal distinct change of metabolites and quality of green tea during the short duration of a single spring season. *J Agric Food Chem*. 2016;64:3302–9 27052744 10.1021/acs.jafc.6b00404

[12] Yu Y, Kou X, Gao R. et al. Glutamine synthetases play a vital role in high accumulation of theanine in tender shoots of albino tea germplasm "Huabai 1". *J Agric Food Chem*. 2021;69:13904–15 34775761 10.1021/acs.jafc.1c04567

[13] Nan C, Joe B. Analysis by high-performance liquid-chromatography of free amino-acids extracted from needles of drought-stressed and shaded *Pinus ponderosa* seedlings. *Physiol Plantarum*. 1990;79:23–30

[14] Li F, Dong C, Yang T. et al. The tea plant CsLHT1 and CsLHT6 transporters take up amino acids, as a nitrogen source, from the soil of organic tea plantations. *Hortic Res*. 2021;8:178 34333546 10.1038/s41438-021-00615-x PMC8325676

[15] Deng W, Fei Y, Wang S. et al. Effect of shade treatment on theanine biosynthesis in *Camellia sinensis* seedlings. *Plant Growth Regul*. 2013;71:295–9

[16] Guo J, Zhu B, Chen Y. et al. Potential 'accelerator' and 'brake' regulation of theanine biosynthesis in tea plant (*Camellia sinensis*). Hortic Res. 2022;9:uhac169 36324642 10.1093/hr/uhac169 PMC9614919

[17] Wen B, Li J, Luo Y. et al. Identification and expression profiling of MYB transcription factors related to L-theanine biosynthesis in *Camellia sinensis*. *Int J Biol Macromol*. 2020;164:4306–17 32861783 10.1016/j.ijbiomac.2020.08.200

[18] Cheng H, Wu W, Liu X. et al. Transcription factor CsWRKY40 regulates L-theanine hydrolysis by activating the *CsPDX2.1* promoter in tea leaves during withering. Hortic Res. 2022;9:uhac025 35184176 10.1093/hr/uhac025 PMC9055099

[19] Wen B, Luo Y, Liu D. et al. The R2R3-MYB transcription factor CsMYB73 negatively regulates L-theanine biosynthesis in tea plants (*Camellia sinensis* L.). *Plant Sci*. 2020;298:110546 32771159 10.1016/j.plantsci.2020.110546

[20] Heerah S, Katari M, Penjor R. et al. WRKY1 mediates transcriptional regulation of light and nitrogen signaling pathways. *Plant Physiol*. 2019;181:1371–88 31409699 10.1104/pp.19.00685 PMC6836853

[21] Rushton P, Somssich I, Ringler P. et al. WRKY transcription factors. *Trends Plant Sci*. 2010;15:247–58 20304701 10.1016/j.tplants.2010.02.006

[22] Teng R, Wang Y, Lin S. et al. *CsWRKY13*, a novel WRKY transcription factor of *Camellia sinensis*, involved in lignin biosynthesis and accumulation. Beverage Plant Res. 2021;1:1–9

[23] Fan X, Yang Y, Li M. et al. Transcriptomics and targeted metabolomics reveal the regulatory network of *Lilium davidii* var. *unicolor* during bulb dormancy release. *Planta*. 2021;254:59 34427790 10.1007/s00425-021-03672-7

[24] Zhao H, Mallano A, Li F. et al. Characterization of *CsWRKY29* and *CsWRKY37* transcription factors and their functional roles in cold tolerance of tea plant. Beverage Plant Res. 2022;2:1–13

[25] Xie Z, Zhang Z, Zou X. et al. Interactions of two abscisic-acid induced WRKY genes in repressing gibberellin signaling in aleurone cells. *Plant J*. 2006;46:231–42 16623886 10.1111/j.1365-313X.2006.02694.x

[26] Zhang Z, Xie Z, Zou X. et al. A rice WRKY gene encodes a transcriptional repressor of the gibberellin signaling pathway in aleurone cells. *Plant Physiol*. 2004;134:1500–13 15047897 10.1104/pp.103.034967 PMC419826

[27] Mauceri A, Abenavoli M, Toppino L. et al. Transcriptomics reveal new insights into molecular regulation of nitrogen use efficiency in *Solanum melongena*. *J Exp Bot*. 2021;72:4237–53 33711100 10.1093/jxb/erab121

[28] Li W, Xiang F, Su Y. et al. Gibberellin increases the bud yield and theanine accumulation in *Camellia sinensis* (L.) Kuntze. *Molecules*. 2021;26:3290 34072521 10.3390/molecules26113290 PMC8198828

[29] Chen W, Zheng C, Yao M. et al. The tea plant *CsWRKY26* promotes drought tolerance in transgenic *Arabidopsis* plants. Beverage Plant Res. 2021;1:1–11

[30] Luo Y, Huang X, Song X. et al. Identification of a WRKY transcriptional activator from *Camellia sinensis* that regulates methylated EGCG biosynthesis. Hortic Res. 2022;9:uhac024 35184160 10.1093/hr/uhac024 PMC9071374

[31] Bulley S, Wilson F, Hedden P. et al. Modification of gibberellin biosynthesis in the grafted apple scion allows control of tree height independent of the rootstock. *Plant Biotechnol J*. 2005;3:215–23 17173621 10.1111/j.1467-7652.2005.00119.x

[32] Anzala F, Paven M, Fournier S. et al. Physiological and molecular aspects of aspartate-derived amino acid metabolism during germination and post-germination growth in two maize genotypes differing in germination efficiency. *J Exp Bot*. 2006;57:645–53 16415333 10.1093/jxb/erj054

[33] El-Yazal M, El-Yazal S, Rady M. Exogenous dormancy-breaking substances positively change endogenous phytohormones and amino acids during dormancy release in 'Anna' apple trees. *Plant Growth Regul*. 2014;72:211–20

[34] Yuan X, Fang R, Zhou K. et al. The *APETALA2* homolog *CaFFN* regulates flowering time in pepper. Hortic Res. 2021;8:208 34719686 10.1038/s41438-021-00643-7 PMC8558333

[35] Ma Y, Xu D, Li L. et al. Jasmonate promotes artemisinin biosynthesis by activating the TCP14-ORA complex in *Artemisia annua*. *Sci Adv*. 2018;4:eaas9357 30627665 10.1126/sciadv.aas9357 PMC6317983

