# Supplementary material for: Gibberellin promotes theanine synthesis by relieving the inhibition of CsWRKY71 on CsTSI in tea plant (Camellia sinensis)
Source: Hortic Res. 2024 Nov 18;12(2):uhae317. doi: 10.1093/hr/uhae317 (PMC11817880; doi:10.1093/hr/uhae317)

Supplementary Information for

**Gibberellin promotes theanine synthesis by relieving the inhibition of CsWRKY71 on *CsTSI* in tea plant (*Camellia sinensis*)**

Fen Xiang^1, 3^, Yi Su^2^, Lingyun Zhou^1, 3^, Cuiting Dai^1, 3^, Xuan Jin^2^, Hongyan Liu^1, 3^, Weigui Luo^4^, Wenbo Yang^1,^ ^3^, Wei Li^1,^ ^2, 3*^

1 Tea Research Institute, Hunan Academy of Agricultural Science, Changsha 410125, China;

2 Hunan Provincial Key Laboratory of Phytohormones and Growth Development, Hunan Agricultural University, Changsha, 410128, China;

3 Hunan Tea Plant and Tea Processing Scientific Observation Experimental Station of the Ministry of Agriculture, Changsha 410125, China;

4 Lushan Botanical Garden, Chinese Academy of Science, Jiu Jiang, 332900, China.

**Email addresses of authors**

Fen Xiang: hncysxf@hunaas.cn

Yi Su: yisu@hunau.edu.cn

Lingyun Zhou: hncyszly@hunaas.cn

Cuiting Dai: hncysdai@hunaas.cn

Xuan Jin: 15770670351@163.com

Hongyan Liu: hncyslhy@hunaas.cn

Weigui Luo: luowg@lsbg.cn

Wenbo Yang: hncysywb@hunaas.cn

Wei Li: hncyslw@hunaas.cn

*Corresponding author. E-mail address: hncyslw@hunaas.cn (Wei Li), Tel.: +86 13574194549, Fax numbers: +86 0731 84690716, Tea Research Institute, Hunan Academy of Agricultural Science, 702 Yuanda Road, Changsha, 410125, P. R. China.

| **Table S1 Primers used in this study** | | | |  |
| --- | --- | --- | --- | --- |
| **Assay** | **Primer name** | **Forward primer (5'to 3')** | **Reverse primer (5' to 3')** | **Restriction Site** |
| *CsTSI* Heterologous transformation | PSN-CsTSI | CTCCGGTTACCCCAAACTCT | ACCGGGGTTCTTTCTCCTTT | BamHI |
| qRT-PCR | CsTSI | ATGAACACAGAGACTTTTTCTACC | GGCTCTTGTTTAAGAAACATGGAG |  |
|  | β-actin (*C. sinensis*) | GCCATCTTTGATTGGAATGG | GGTGCCACAACCTTGATCTT |  |
|  | GFP | AGAGGGTGAAGGTGATGCAA | ACGTGTCTTGTAGTTCCCGT |  |
|  | β-actin (*A. thaliana*) | GCCATCTTTGATTGGAATGG | GGTGCCACAACCTTGATCTT |  |
|  | CsWRKY71 | CTCCGGTTACCCCAAACTCT | ACCGGGGTTCTTTCTCCTTT |  |
|  | CsERF11 | AACGGCAAGGAAACTCGCTA | ACGACGGTGATCTTGTTGCT |  |
|  | CsPBF1 | CAGTTTTGTTCGGCCTCTCC | ACCGAAGAAATGGGCCAAAC |  |
| *proCsTSI* Heterologous transformation | pC1302-proCsTSI | GCAGGCATGCAAGCTTATAGTTGGATGTAATGTTTTGTTTGTT | CTCAGATCTACCATGGTTCTCTCAGCTCTGCAAATTTCTCC | HindIII /NcoI |
| Y1H | His-proCsTSI | GTAATACGACTCACTATAGGGCGAATTCTATTTAAAATTACTCAAAAGGGAGATAAACT | CGATTCGCGAACGCGTGAGCTCCTCTCTCTCTCTCTCTACGATCAAAACT | ECORI/SacI |
|  | pGADT7-CsWRKY71 | CATGCATATGTCTGATGAACACAGAGACTTTTTCTA | CATGGGATCCTCATGGCTCTTGTTTAAGAAACATGG | NdeI/BamHI |
| EMSA | His-WRKY71 | CATGCATATGTCTGATGAACACAGAGACTTTTTCTACC | CATGCTCGAGTCATGGCTCTTGTTTAAGAAACATGGAG | NdeI/XhoI |
| Dual-luciferase transient expression assay | WRKY71-62sk | CGGCCGCTCTAGAACTAGTGGATCCATGTCTGATGAACACAGA | TATCGATAAGCTTGATATCGAATTCTCATGGCTCTTGTTTA | BamHI/EcoRI |
|  | proCsTSI-0800 | TAAGCTTGATATCGAATTCCTGCAGATCCTATTTAAAATTACTC | CGGCCGCTCTAGAACTAGTGGATCCATCCCTCTCTCTCTCT | SalI/HindIII |
| antisense oligodeoxy nucleotides interfering assay | sOND-CsWRKY71 | AGAAAAAGGAGAAAGAGCCC |  |  |
|  | asOND-CsWRKY71 | AAGAGTTGTGAAAGCCTTGC |  |  |
| Subcellular localization | 35S::WRKY71-EGFP/PHBT | GAATTCTGCAGTCGACCATATGTCTGATGAACACAGAGACT | ATGGATCCCGGGCGCCGCGGTGGCTCTTGTTTAAGAAACATGGAG | SalI/SacII |

**Table S2 *cis*-acting regulatory elements analysis of *CsTSI* promoter sequences**

| Factor or Site Name | Location | Number | Sequence |
| --- | --- | --- | --- |
| TATABOXOSPAL | -1350 | 1 | TATTTAA |
| CACTFTPPCA1 | -1339, et al | 25 | YACT |
| **PBF/PYRIMIDINEBOXOSRAMY1A** | **-1334, et al** | **3** | **CCTTTT** |
| DOFCOREZM | -1333, et al | 26 | AAAG |
| GATABOX | -1326, et al | 12 | GATA |
| GT1CONSENSUS | -1326, et al | 19 | GRWAAW |
| IBOXCORE | -1326, et al | 4 | GATAA |
| TAAAGSTKST1 | -1315, et al | 5 | TAAAG |
| NODCON2GM | -1289, et al | 6 | CTCTT |
| EECCRCAH1 | -1284, et al | 5 | GANTTNC |
| ERELEE4 | -1275 | 1 | AWTTCAAA |
| CAATBOX1 | -1260, et al | 17 | CAAT, CAATT, CCAAT,CAAAT, TGCCAAC |
| TBOXATGAPB | -1242, et al | 2 | ACTTTG |
| CARGCW8GAT | -1229, et al | 8 | CWWWWWWWWG |
| POLASIG | -1226, et al | 22 | AATAAA, AATAAT, AATTAAA, AATAAG |
| NODCON1GM | -1225, et al | 6 | AAAGAT |
| PRECONSCRHSP70A | -1212, et al | 3 | SCGAYNRNNNNNNNNNNNNNNNHD |
| PYRIMIDINEBOXHVEPB1 | -1210 | 1 | TTTTTTCC |
| GT1GMSCAM4 | -1209, et al | 8 | GAAAAA |
| CIACADIANLELHC | -1200 | 1 | CAANNNNATC |
| REBETALGLHCB21 | -1191 | 1 | CGGATA |
| MYBST1 | -1190, et al | 4 | GGATA |
| TATABOX | -1184, et al | 16 | TATAAAT, TATTAAT, TATATAA, TTATTT, TACAAAA, TTTTA, TAATA, TATA, TTTTA, TACATAAA, TATAAAT, ATATAT |
| ROOTMOTIFTAPOX1 | -1163, et al | 15 | ATATT |
| ANAERO1CONSENSUS | -1159, et al | 2 | AAACAAA |
| INRNTPSADB | -1149, et al | 7 | YTCANTYY |
| -300CORE | -1106 | 1 | TGTAAAG |
| -300ELEMENT | -1106, et al | 4 | TGHAAARK |
| NTBBF1ARROLB | -1104, et al | 2 | TGTAAAGT |
| SEF4MOTIFGM7S | -1080, et al | 6 | RTTTTTR |
| CURECORECR | -1068, et al | 14 | GTAC |
| ARFAT | -958 | 1 | TGTCTC |
| SURECOREATSULTR11 | -958, et al | 4 | GAGAC |
| SREATMSD | -936, et al | 2 | TTATCC |
| -10PEHVPSBD | -911, et al | 3 | TATTCT |
| MYBPLANT | -660, et al | 2 | MACCWAMC |
| MYB1AT | -657, et al | 3 | WAACCA |
| WBOXATNPR1 | -653 | 1 | TTGAC |
| **WRKY71OS** | **-510, et al** | **2** | **TGAC** |
| PALBOXAPC | -634, et al | 2 | CCGTCC |
| CARGNCAT | -629 | 1 | CCWWWWWWWWGG |
| RBCSCONSENSUS | -583, et al | 2 | CAATWATTG |
| CPBCSPOR | -417, et al | 2 | TATTAG |
| EBOXBNNAPA | -229, et al | 4 | CANNTG |
| SP8BFIBSP8BIB | -195 | 1 | TACTATT |
| LTREATLTI78 | -187 | 1 | ACCGACA |
| WBOXNTERF3 | -55 | 1 | TGACY |

**Table S3 Correlations between transcription factors and *CsTSI* expression in tea leaves.**

|  | CsWRKY71 | CsERF11 | CsPBF1 |
| --- | --- | --- | --- |
| CsTSI | -0.96** | 0.83* | -0.76* |

*and **, represent statistical t-test *P values* less than significance levels 0.05, 0.01 respectively.

**Figure S1 Biological function verification of *CsTSI* in BJ1.** **a**, **b**. GUS expression in leaves of col-0 strain. **c**, **d**. GUS expression in roots of *col-0* strain. **e**, **f**. GUS expression in leaves of *CsTSI::GUS* strain. **g**, **h**. GUS expression in roots of *CsTSI::GUS* strain. **i**. Identification of biological functions of *CsTSI* by feeding *CsTSI::GUS* strain with theanine synthesis substrate ethylamine (EA).


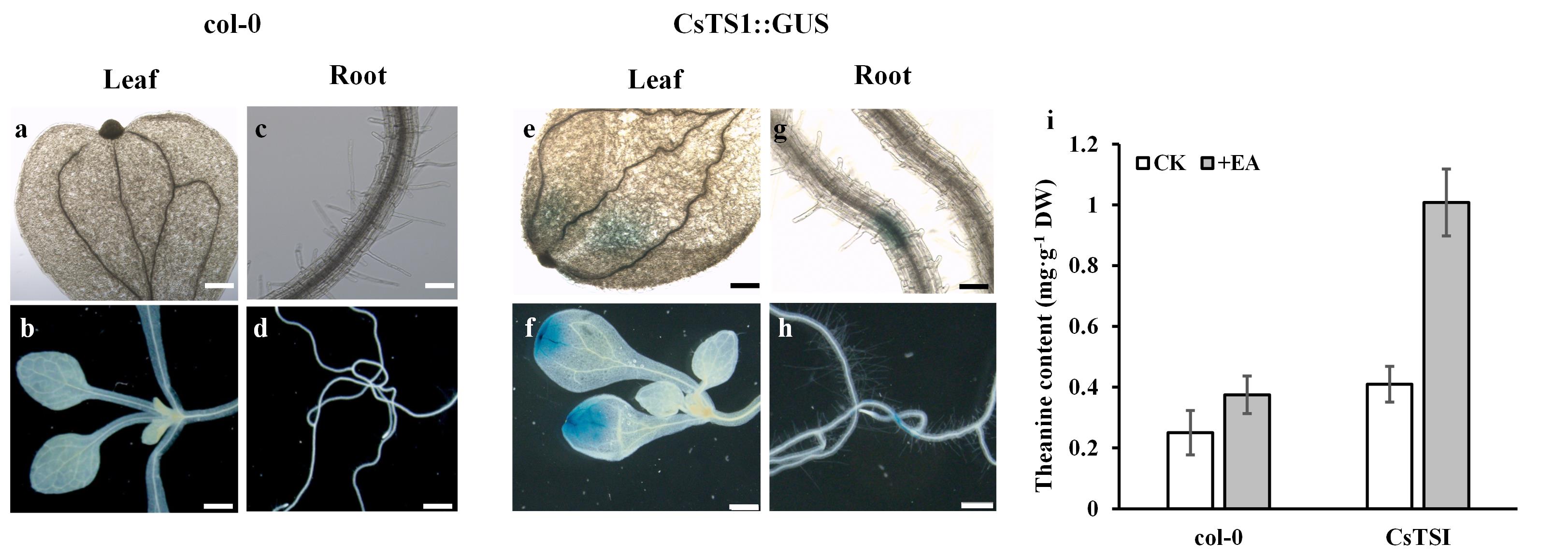


**Figure S2 Screening and analysis of transcription factors related to gibberellin signal transduction pathway** **in *C. sinensis* after GA treatments.** **a**, Pie chart of up and down regulated DEGs; **b**, Cluster heat map of DEGs; **c–f**, Determination of the relative expression of *CsTSI* (c) and transcription factors related to gibberellin signal transduction pathway (d–f) by qPCR.


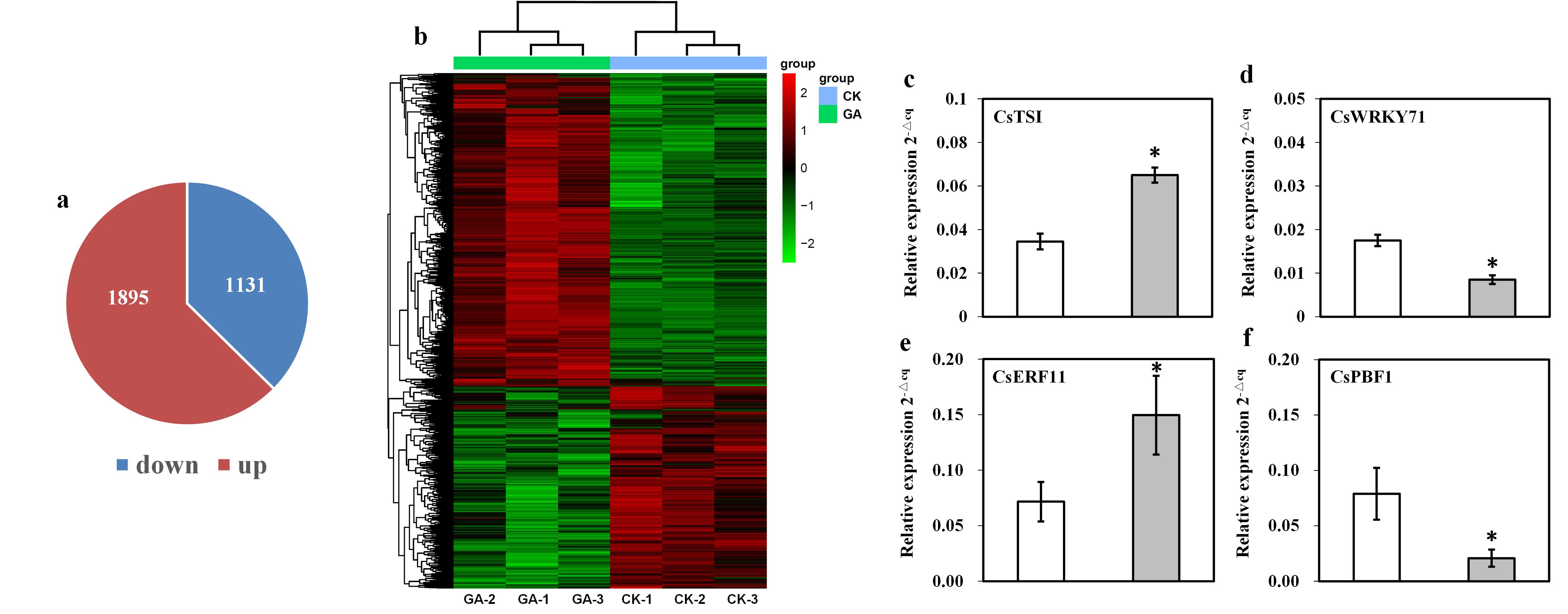

Supplement: Web_Material_uhae317 [file web_material_uhae317.docx]
